# Control of the Bone Morphogenetic Protein 7 Gene in Developmental and Adult Life

**DOI:** 10.2174/138920209788488490

**Published:** 2009-06

**Authors:** Leif Oxburgh

**Affiliations:** Center for Molecular Medicine, Maine Medical Center Research Institute, 81 Research Drive, Scarborough, ME 04074, USA

**Keywords:** BMP, BMP7, bone morphogenetic protein, gene regulation, enhancer element.

## Abstract

The TGFβ superfamily growth factor BMP7 performs essential biological functions in embryonic development and regeneration of injured tissue in the adult. *BMP7* activity is regulated at numerous levels in the signaling pathway by the expression of extracellular antagonists, decoy receptors and inhibitory cell signaling components. Additionally, expression of the *BMP7* gene is tightly controlled both during embryonic development and adult life. In this review, the current status of work on regulation of *BMP7* at the genomic level is discussed. *In situ* hybridization and reporter gene studies have conclusively defined patterns of *BMP7* expression in many tissues. Additionally, both *in vivo* and cell culture studies have defined some of the mechanistic bases for this regulation. In addition to transcriptional activation mediated by binding of activating transcription factors, there is also strong evidence for repression through recruitment of histone modifying enzymes to specific genetic elements. This review summarizes our current understanding of *BMP7* gene regulation in embryonic development and adult tissues.

## BMP7 IN DEVELOPMENT AND DISEASE

The bone morphogenetic proteins (BMPs) were first identified as a group of osteoinductive proteins isolated from bovine bone [1]. Subsequent studies have shown that BMPs function as regulators of diverse developmental processes. Inactivation of *Bmp7* reveals requirements for this gene in development of the kidney, eye, hindlimb [2, 3], lacrimal gland [4], and brown adipose tissue [5]. Compound inactivation with other *Bmp*s or BMP modulators demonstrates additional functions in heart development [6], ventral mesoderm specification [7], and limb patterning [8]. As the *Bmp7* null mutant is not viable, functions in the adult have been characterized largely by *in vitro* experiments. In the kidney, BMP7 is thought to prevent and perhaps even reverse the epithelium to mesenchyme transition associated with fibrotic conditions [9, 10], and counteract diabetic nephropathy [11, 12]. BMP7 also accelerates bone differentiation and fracture healing [13, 14], a feature that is currently being therapeutically exploited.

## OVERVIEW OF BMP SIGNALING

BMPs belong to the Transforming Growth Factor β (TGFβ) superfamily of cytokines that regulate an array of fundamental cellular processes, including proliferation, differentiation and cell death. Signaling is initiated when ligands bind cell surface serine threonine kinase BMP receptors (BMPRs). A complex of type I and type II receptors is formed, in which the constitutively active BMPRII phosphorylates BMPRI, which in turn phosphorylates the receptor associated transcription factors SMAD 1, 5 and 8 (R-SMADs) [15, 16]. Phosphorylated R-SMADs associate with the common SMAD, SMAD4 [17] and are translocated into the nucleus where they bind DNA in association with other factors. Transcriptional outcomes are determined by association with general transcriptional activators such as p300 and CBP [18], or by association with repressors such as Suv39h [19] or CtBP [20]. In addition to activating SMAD-dependent intracellular signaling, BMPs also activate p38 and JNK pathways through BMP-receptor associated TGFβ activated kinase 1 (TAK1) [21-23]. Regulation of the balance between activation of these two distinct intracellular cascades is incompletely understood. Essential signaling components such as SMADs [24] and TAK1 [21, 25] are differentially expressed, influencing the potential for activation of these signaling cascades by BMPs in distinct cell types. Additionally, it has been shown that the mode of physical interaction between BMPRI and BMPRII receptors influences signaling outcome following ligand binding. Activation of preformed BMPRI/RII complexes at the cell surface results in SMAD activation, whereas ligand-induced assembly of BMPRI/RII complexes preferentially results in MAPK activation [26]. Factors that differentially regulate the mode of receptor complex formation have not yet been identified. Although SMAD-dependent and SMAD-independent signaling pathways promote distinct transcriptional outcomes, it appears that these intracellular cascades function cooperatively in numerous contexts [21, 27-29].

## THE BMP LIGANDS

In the mouse, 17 distinct proteins are classified as BMPs [30]. Of these, the BMP 2, 4, 5, 6 and 7 signaling ligands have been most extensively studied. On the basis of sequence similarity with prototypic Drosophila family members, vertebrate BMPs can be divided into two sub-groups, which share approximately 30% identity at the amino acid level. The vertebrate BMP2 and BMP4 genes are functional orthologues of Drosophila Decapentaplegic (Dpp), whereas the vertebrate BMPs 5, 6 and 7 and the fly ligand Glass bottom boat (Gbb) constitute a distinct sub-group. The conservation of these separate BMP sub-groups across a wide phylogenetic distance suggests that dpp/BMP2/BMP4 and gbb/BMP5/BMP6/BMP7 may be functionally distinct. In Drosophila development, a balance between Dpp and Gbb is required for morphogenesis of the wing imaginal disk [31], indicating differential biological effects of these two ligands. Functional divergence of the two ligands associates with specificities for distinct receptors: Dpp primarily signals through the Thickveins (Tkv) receptor, whereas Gbb signals through both the Tkv and the Saxophone (Sax) receptors [32]. The combinatorial signaling outcome displays a counterintuitive complexity, as Sax functions both as a positive mediator of Gbb signaling, and also as a negative regulator, sequestering this ligand and preventing it from signaling through Tkv [33]. Although the relationships between ligands and receptors in the mammalian signaling system is more difficult to interpret due to extensive redundancy, ligand-receptor interactions do display differential affinities, indicating that similar combinatorial interactions may regulate ligand interpretation by cells. Specifically, BMP4 binds the BMPRIs ALK3 and ALK6 strongly, whereas BMP7 efficiently binds the BMPRIs ALK6 and ALK2 [34, 35]. ALK2 is the ortholog of Sax, and it is therefore tempting to speculate that it may function similarly as both a signal transducer and antagonist. Signaling output from the receptor does differ depending on the identity of the associated BMPRII receptor [35]. However, combinatorial inactivation of receptors in mesenchymal stem cells using an RNAi approach does not reveal an antagonistic effect of ALK2 in combination with any BMPRII receptor, arguing against conservation of the Dpp/TKV versus Gbb/Sax paradigm, at least in this cellular context [36]. A genetic test of this hypothesis has been conducted in the mouse by conditional inactivation of *Alk2* and *Alk3* either separately or in combination in the mouse Müllerian duct [37]. BMP signaling is required for Müllerian duct involution in the male, and provides a sensitive genetic system with which to assay signal transduction *in vivo*. Interestingly, the two BMPRI receptors promote BMP signaling in a redundant fashion, again arguing against any inhibitory activity of BMP5/6/7/ALK2. Despite the interesting inference based on comparison with signaling in Drosophila, little concrete evidence of differing biological outcomes of distinct BMP-receptor pairings has been found in mammalian cells.

## SHARED VERSUS UNIQUE BIOLOGICAL PROPERTIES OF BMP7

To try to ascertain whether BMP ligands have distinct *in vivo* functions, a genetic complementation strategy has been used to functionally compare *Bmp6*, *Bmp7*, and *Bmp4 *[38]. The analysis was performed by comparing the potential of *Bmp6 *and* Bmp4 *to rescue embryonic kidney development in the *Bmp7* null mouse strain. Both the closely related *Bmp6*, and the more distantly related *Bmp4* are capable of substituting for *Bmp7* in early kidney development, suggesting that distinct BMPs may be functionally interchangeable *in vivo *in this context. However, *Bmp7* null mice expressing *Bmp4* from the *Bmp7* locus are not viable, and it therefore remains likely that there also are unique functions of these ligands that are required for embryonic development. Functional differences between BMPs other than the above mentioned differences in receptor affinities have been demonstrated *in vitro*. The extracellular protein antagonist Noggin binds BMP4 with higher affinity than BMP7 [39]. Conversely, the antagonist Chordin-like 1 inactivates BMP7, but not BMP4 [40]. These antagonists are differentially expressed during embryonic development and in the adult, potentially creating zones of activity of various BMPs. One consequence of reducing availability of ligand may be to influence the balance between SMAD-dependent and SMAD-independent signaling. Comparison of intracellular signaling promoted by varying concentrations of BMP7 reveal preferential activation of p38 MAPK signaling at low concentrations, and phosphorylated SMAD1/5/8 at high concentrations in collecting duct cells and proximal tubule cells of the adult kidney [41, 42]. A unique function has furthermore been proposed for BMP7 in counteracting, and even reversing epithelial to mesenchymal transition caused by TGFβ in epithelial cells of the kidney by activating the E-cadherin gene [9, 43]. The signaling mechanism underlying this phenomenon has not yet been clarified, and remains puzzling since the activity is unique to BMP7 and cannot be reproduced with the closely related ligand BMP6 [9]. The feature may be unique to cell types used in the study however, as other authors have been unable to reproduce the result in primary human proximal tubule cells [44].

A model consistent with data at hand would be that BMPs 5, 6 and 7 derive from a common ancestral BMP, are functionally interchangeable, and have evolved distinct partially overlapping domains of expression through addition of unique genomic regulatory elements. Consistent with this idea, individual inactivation of these genes reveals phenotypes that closely correspond with their domains of unique expression [2, 3, 45-47]. Furthermore, highly exacerbated phenotypes are revealed by compound mutation of the *Bmp5*, *Bmp6* and *Bmp7* genes, [6, 48], indicating that the collective expression of these ligands determines developmental function in domains of overlapping expression.

## REGULATION OF THE GENE ENCODING BMP7

### BMP7 in the Mouse Genome

**Fig. (1):**
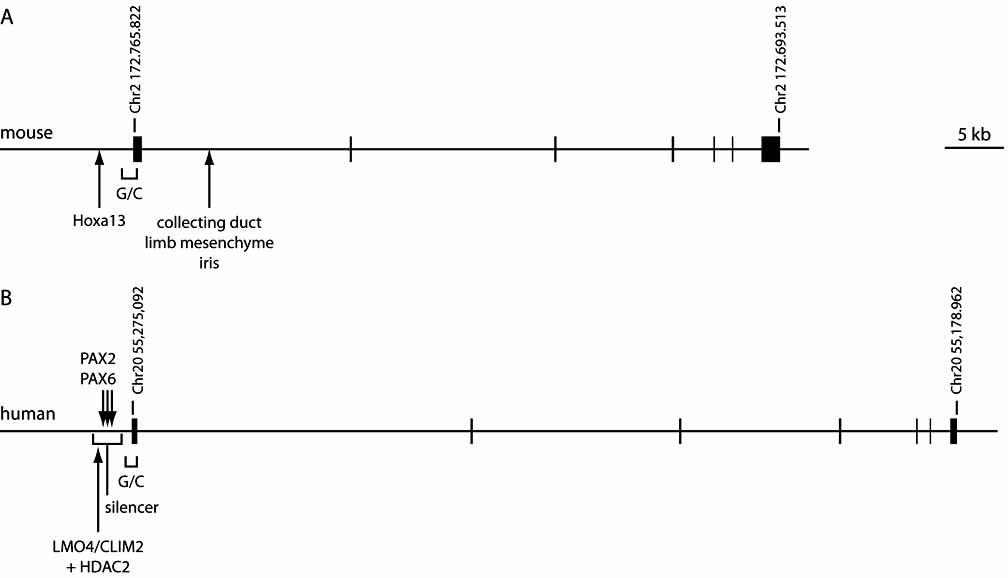
**Organization of *BMP7* loci. A.** Schematic of mouse *Bmp7* depicting the functionally verified HOXA11 binding site, the G/C rich region surrounding the transcriptional initiation site, and the *in vivo* validated enhancer island controlling expression in kidney collecting duct, limb mesenchyme, and iris. **B.** Schematic of human *BMP7* showing the approximate locations of the silencer element, the LMO4/Clim2 binding site, PAX2/6 binding sites, and the G/C rich region surrounding the promoter. Enhancer and repressor elements in the human locus have been identified and validated *in vitro.*

In the mouse,* Bmp7* is composed of 7 exons located distally on chromosome 2 (Fig. **1A**). This region is thought to contain a number of imprinted genes [49]. However, expression analysis shows that *Bmp7* is expressed from both maternal and paternal alleles in weanling mice, indicating that the gene is not imprinted [49]. This analysis was conducted on kidney tissue, and it remains possible that imprinting occurs in other tissues. Two different Northern analyses have each revealed transcription of four separate splice forms of *Bmp7* in the embryonic mouse kidney, however the precise sizes of these transcripts are not in complete agreement. In one study, transcripts of 1.8 kb, 2.1 kb, 2.4 kb and 4 kb were identified [50], whereas in another, 1.9 kb, 2.1 kb, 3.6 kb and 3.8 kb transcripts were detected [51]. These distinct transcripts all contain identical coding sequence but vary in their 5’ and 3’ untranslated regions (UTRs) because of the use of two independent initiation sites and two termination sites [51]. Transcriptional initiation sites have been identified, but do not conform to the TATA/CAAT transcriptional initiation paradigm [51, 52]. They are located within a GC rich region that serves as a core promoter (Fig. **1A**). Initiation from TATA-less promoters is generally not precisely regulated [53], providing a likely explanation for the prevalence of distinct initiation sites in *Bmp7*. Functional consequences of alternate polyadenylation have not been reported for *Bmp7*. However, comparison of RNA by Northern blot from adrenal and kidney of newborn mice shows that long isoforms predominate in the adrenal, but both isoforms are almost equally represented in the kidney [50], indicating that there may be tissue-specific polyadenylation site preferences.

**Fig. (2):**
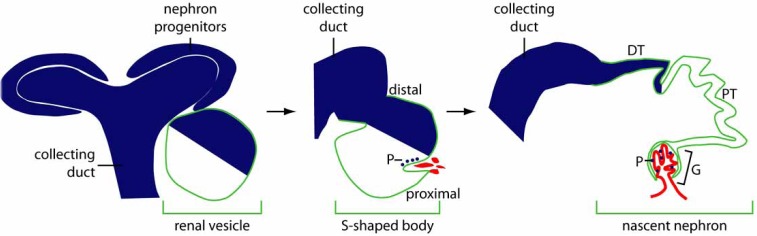
**Cell type specific control of Bmp7 transcription in the developing kidney.** At early stages of kidney development, *Bmp7* is expressed in both the collecting duct and nephron progenitors. Upon induction of progenitors to form a renal vesicle, *Bmp7* expression is regionalized, and is limited to the portion of the renal vesicle fated to form the distal nephron. As the renal vesicle progresses to the S-shaped body stage, distal expression of *Bmp7* is maintained, and a small population of presumptive podocyte cells adjacent to the endothelial cleft at the proximal pole of the developing nephron also express *Bmp7*. This expression pattern is maintained throughout growth of the nephron, and the complex morphogenesis of the glomerulus. **Abbreviations:** DT, distal tubule; G, glomerulus; P, podocyte, PT, proximal tubule.

Expression of *Bmp7* is strictly transcriptionally regulated both during development, and in the adult. Early studies employing *in situ* hybridization revealed that although *Bmp7* is expressed in most organ systems, the pattern of expression within each organ system is intricately regulated [47, 54, 55]. The development of a reporter mouse strain expressing β-galactosidase from the *Bmp7* locus has facilitated more spatially precise expression analysis [56]. Transcription of *Bmp7* has been most comprehensively studied in the kidney where gene expression has been mapped in detail from the earliest stages of development to the adult (Fig. **2**). The permanent kidney of the mouse develops from two major cell lineages, which are both derived from intermediate mesoderm. The nephron lineage forms all segments of the nephron, or filtering unit, while the collecting duct lineage is fated to become the collecting system of the adult kidney [57-60]. *Bmp7* is expressed in collecting ducts throughout development [47, 56], and remains actively expressed in collecting ducts of the adult [61]. In contrast, expression in the nephron lineage is highly dynamically regulated: although progenitor cells of the developing nephron uniformly express *Bmp7*, only 2 specific cell types within the nephrons derived from these cells express the gene: podocytes of the glomerulus, and distal tubule cells [56, 61]. Whether *Bmp7* expression is inactivated upon differentiation of nephron progenitors, and subsequently reactivated in these differentiated cell types, or whether it is maintained in a subset of designated progenitors throughout their differentiation is unclear. Other organ systems have similarly complex patterns of expression, possibly reflecting the evolution of BMP7 as a morphogen, with separate populations producing the ligand, and others receiving it.

Regulation of *Bmp7* expression has been studied in the developing limb and kidney. Screening of the region surrounding exon 1 using transgenic reporter mice reveals tissue specific regulatory elements for expression in eye, kidney, central nervous system and limb [51], encompassing a significant proportion of all developmental expression of *Bmp7*. Closer analysis of expression in the kidney reveals that enhancer elements driving expression in nephron progenitor cells and in collecting duct cells are spatially separate. The collecting duct enhancer is located in intron 1 of the gene, and mutational analysis reveals that it corresponds to a FOXD3 binding site. Since FOXD3 expression is limited to neural crest during development, it is most likely that a transcription factor with a similar binding site is activating *Bmp7* transcription in kidney collecting ducts. In addition to the collecting duct, the FOXD3 binding site also regulates gene expression in specific domains of the limb and eye, and can be found within an approximately 500 bp enhancer island that displays 80% identity at the nucleotide level with genomes as evolutionarily distant as Xenopus tropicalis. Analysis of the remaining locus does not reveal any further islands of intronic sequence over 20 base pairs in length that are conserved to a similar degree with X. tropicalis. A question that remains to be explored is whether additional regulatory sequence is located distant from the locus, as has been observed for the *Bmp4* and *Bmp5* genes [62, 63]. Close scrutiny of expression in the developing limb was prompted by studies of a mouse strain null for the gene encoding the HOXA13 transcription factor. This mutant strain displays defects in separation of the digits, a process that BMP signaling is known to regulate [64, 65]. *Hoxa13*, *Bmp2* and *Bmp7* are expressed in an overlapping manner in interdigital mesenchyme, and disruption of *Hoxa13* significantly reduces both *Bmp2* and *Bmp7 *expression [66]. Using a synthetic peptide mimicking the DNA binding activity of HOXA13, an enhancer element was located approximately 3 kb upstream of the promoter region. HOXA13 binding at this site was confirmed by chromatin immunoprecipitation [66]. Cell type specific regulation of *Bmp7* by HOXA13 may occur in additional tissues, as dysregulation of *Bmp7 *has also been observed in the genitourinary tract of *Hoxa13* mutant mice [67].

Studies of regenerating adult mouse kidneys recovering from ischemically induced experimental injury show an increased overall level of histone acetylation relative to uninjured controls, with a concomitant reduction in histone deacetylase 5 (HDAC5) expression [68]. Interestingly, siRNA mediated knock down of HDAC5 increases *Bmp7* expression in the rat kidney epithelial cell line NRK-52, indicating that the gene may normally be repressed through histone modification, and that this might be reversed in the regenerative response. Studies of the dynamics of *Bmp7* expression in the kidney following acute ischemia have yielded conflicting results, with Northern analyses demonstrating a significant decrease by 48 hours post ischemia, and a return to baseline levels approximately 7 days after injury [69]. Immunohistochemical studies on the other hand show a substantial increase 48 hours post ischemia [70]. This discrepancy does not appear to reflect analysis of different regions of the kidney in these two studies, as both focused on the medulla. Further inquiry will be required to fully characterize *Bmp7* expression in the regenerative response, and to understand whether histone acetylation is a mechanism to promote production of reparative growth factors such as BMP7 in injured tissue.

### BMP7 in the Human Genome

**Fig. (3):**
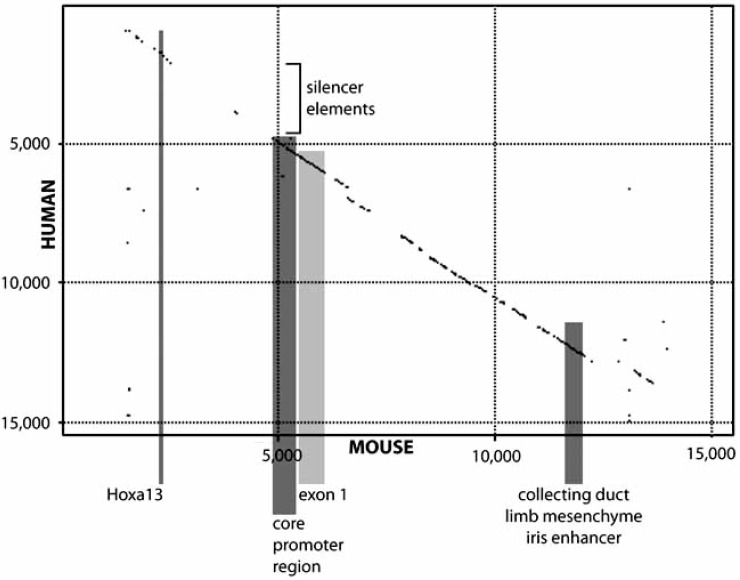
**Human-mouse comparison of the region surrounding exon 1.** Comparison using a window size of 30 nucleotides and a minimum identity of 60% reveals extensive conservation between mouse (x-axis) and human (y-axis) loci. Regions of specific interest are shaded: the regulatory elements are shaded dark grey, and exon 1 is shaded light grey. Although there is a high level of sequence conservation between species at these sites, there is little or no conservation within the region containing silencer elements identified in the human gene.

*BMP7* is located on chromosome 20 [71]. Two BMP7 transcripts of approximately 2.2 kb and 4 kb can be detected in a variety of fetal tissues [55], and in cultured kidney cells [72] (Fig. **1B**). Library screening of cDNA derived from polyadenylated transcripts identifies one approximately 2 kb transcript [73]. Subsequent analysis of cDNA sequences deposited at NCBI identify an additional 4 kb transcript with identical coding sequence, but longer 3’ UTR (NCBI accession number NM_001719). Promoter analysis reveals a single transcriptional initiation site [74], and it can therefore be concluded that the difference in molecular weight between the two prevalent mRNA species is due to the usage of alternate polyadenylation sites. Comparison with the mouse reveals approximately 75% identity in the 750 bp immediately preceding the transcriptional initiation site, a similar GC-rich nucleotide composition and a lack of TATA/CAAT motifs [74] (Fig. **3**). Deletion analysis demonstrates the presence of a core promoter spanning approximately 600 bp of sequence immediately upstream of the transcriptional initiation site, and also shows that the activity of this promoter is strongly negatively regulated by silencer elements immediately upstream [74, 75]. Comparison of *BMP7 *promoter activities in osteosarcoma Hos cell cells and kidney derived Cos-7 cells verifies the presence of silencer elements, but shows that their usage is cell type specific, with Hos cells demonstrating a strong silencing effect [75]. Analysis of the *BMP7* promoter in mammary tumor epithelial cells reveals binding of the LIM-only protein 4 (LMO4) within the silencer region [76]. LMO4 is a non-DNA binding protein that associates with DNA through interaction with other transcription factors. By combining chromatin immunoprecipitation with the use of a doxycycline-regulated LMO4-expressing cell line, the authors were able to show that low level binding of LMO4 in complex with the transcription factor Clim2 in the basal, non-activated state promotes recruitment of histone deacetylase 2 (HDAC2), thus potentially silencing transcription through histone modification. Extensive binding of LMO4/Clim2 in the activated state instead reduces recruitment of HDAC2, thus potentially relieving transcriptional silencing.

As in the mouse, tissue and cell type specific regulation of *BMP7* is best understood in the kidney. At 6.5 weeks of development, intense *BMP7* expression can be seen in nephron progenitors and collecting ducts, while at 10 weeks expression has resolved to podocytes of the glomeruli, and regions of the tubular nephron. The latter have not been defined by marker analysis as distal tubules, but in analogy with the mouse, it seems highly likely that it is the distal nephron segment that expresses *BMP7* [55]. Indeed, immunohistochemical localization of BMP7 in the adult confirms protein expression in the distal tubule. Surprisingly, protein cannot be identified in adult human podocytes [77]. This may reflect a genuine species difference between mouse and human, but is perhaps more likely to reflect a lack of *BMP7* translation in the podocyte as both species demonstrate intense mRNA expression in this cell type during late gestation. Regulation of cell type specific transcription is less well defined *in vivo* for human *BMP7* than for the mouse ortholog, but studies have identified regulatory circuits in cultured cells. Deletion analysis has determined that both PAX2 and PAX6 function as activators of *BMP7* transcription in *in vitro* transcriptional activation assays of the genomic region immediately upstream of the core promoter [74]. This is potentially important for the understanding of transcriptional regulation in the kidney as the PAX2 transcription factor is absolutely required for normal kidney development [78-80], and is expressed in an overlapping manner with *Bmp7* both in nephron progenitor cells and collecting duct [81]. PAX2 is thus a strong candidate regulator of *BMP7* expression in nephron progenitors.

## COMPARATIVE ASPECTS

As anticipated, extensive conservation is seen between mouse and human *BMP7* loci. A dot-plot of nucleotide sequence identity in 15 kb of sequence surrounding the first exon demonstrates regions of approximately 80% identity for the Hoxa11 binding site, the core promoter, and the intronic enhancer island regulating expression in kidney collecting duct, limb mesenchyme and iris (Fig. **3**). *BMP7* may thus largely be controlled by similar transcriptional circuitry in the two species. One notable exception, however, is the silencer region preceding the core promoter of human *BMP7*. Interestingly, this region is almost completely divergent in the mouse upstream region. To ascertain whether this silencer region is located elsewhere within the mouse *Bmp7* locus, dot plots comparing the human silencer region with the entire *Bmp7* locus including all upstream and downstream intergenic sequence were generated. No region of greater than 30 nucleotides with 60% or better identity could be located on either strand, and it thus appears that this regulatory element is absent in mouse *Bmp7*. The finding that the silencer element is utilized in Hos ostesarcoma and G401 rhabdoid kidney tumor cell lines, but not in normal transformed Cos-7 kidney cells [74, 75] demonstrates cell-type specificity and argues that it is not part of the core promoter sequence. The association of this region with LMO4/Clim2 and histone deacetylase in human mammary tumor epithelial cells [76] furthermore provides a molecular mechanism for transcriptional control of the gene by the silencer element. However, the paucity of knowledge regarding *in vivo* gene regulation of human* BMP7* precludes prediction of the role of this regulatory sequence in embryonic development or homeostasis of the adult. Further studies comparing the regulatory function of the silencer between cell lines derived from normal human tissue are necessary to better understand cell-type specific control of *BMP7* by this fascinating element.

## FUTURE PERSPECTIVES

Although relatively few studies have analyzed genomic regulation of the *BMP7* gene, a complex and interesting picture is emerging. Studies using genetically modified mice to map the regulatory functions of non-coding sequence are labor intensive, but have been highly informative. The availability of genome sequences from multiple species now allow for high resolution *in silico* prediction of regulatory sequence by species comparison. Identification of fewer candidate elements with a higher degree of confidence will speed *in vivo* validation, and enable a more global understanding of transcriptional networks controlling *Bmp7* in specific cells and tissues.

## ABBREVIATIONS

BMP: = Bone Morphogenetic Protein
BMPR: = BMP receptor
bp: = Base pairs
HDAC: = Histone deacetylase
kb: = Kilobases
R-SMAD: = Receptor associated Smad
TAK1: = TGFβ associated kinase 1
TGFβ: = Transforming Growth Factor β
UTR: = Untranslated region
